# Nursing assistants’ knowledge, attitudes and training needs regarding urinary incontinence in nursing homes: a mixed-methods study

**DOI:** 10.1186/s12877-023-03762-z

**Published:** 2023-01-22

**Authors:** Lulu Liao, Hui Feng, Jingjing Jiao, Yinan Zhao, Hongting Ning

**Affiliations:** 1 School of Nursing, Tongji Medical College, Huazhong University of Science and Technology, Wuhan, China; 2 Xiangya School of Nursing, Central South University, Changsha, China; 3 Xiangya-Oceanwide Health Management Research Institute, Central South University, Changsha, China; 4 National Clinical Research Center for Geriatric Disorders, Xiangya Hospital, Changsha, China

**Keywords:** Urinary incontinence, Training, Nursing assistants, Nursing homes, Mixed methods

## Abstract

**Background:**

Urinary incontinence is an increasingly common problem, especially among older people in nursing homes. Nursing assistants are the leading workforce in nursing homes, and their knowledge and attitudes regarding urinary incontinence have garnered considerable attention in the context of aging in China. However, most previous studies on this issue have focused on registered nurses. This study aimed to explore nursing assistants’ knowledge, attitudes and training needs with regard to urinary incontinence.

**Methods:**

We conducted a two-part mixed-methods study. After institutional manager approval, we surveyed the knowledge and attitudes of 509 nursing assistants regarding urinary incontinence. We carried out semi-structured interviews with 40 nursing assistants to elicit detailed information on training needs.

**Results:**

In general, knowledge about urinary incontinence was poor (14.00 ± 4.18), although attitudes were primarily positive (35.51 ± 3.19). Most nursing assistants were very willing to learn more about urinary incontinence (93.9%, 478/509), but time constraints and low educational background may be barriers to learning motivation. The three preferred training styles among nursing assistants were face-to-face guidance from a mentor, training combining theory with practice, and online video training.

**Conclusions:**

Chinese nursing assistants had poor knowledge but positive attitudes toward urinary incontinence. Facility managers should focus on developing training and learning mechanisms regarding urinary incontinence. It is important to adopt diverse training styles according to the actual situation of nursing homes.

## Background

Urinary incontinence (UI), which is defined as the uncontrolled leakage of urine, has a high prevalence in the older population and among nursing home residents in particular [1]. In 2021, the prevalence of UI was approximately 24.3% among older people aged ≥75 years in Chinese nursing homes (NHs) [2]. The prevalence of UI increased from 12.0% for men aged 70–74 years old to 16.3% for those aged 90 years and older [3]. The incidence of UI in post-menopausal and middle-aged women ranges from 44 to 57% [4]. UI is generally experienced as shameful and repulsive, making it challenging for people experiencing UI to seek health care services [5].

Previous studies have indicated that UI might contribute to several adverse consequences in older people such as (depression, anxiety, low socialization and high cost) [6, 7]. Untreated incontinence can lead to dermatologic complications, such as incontinence dermatitis, intertrigo, and urinary tract infection [8]. Furthermore, there is a higher mortality rate among individuals with UI than among those without UI [9]. Thus, the prevention and management of UI and its complications have been identified as a priority for quality improvement (QI) [10].

In Chinese NHs, registered nurses make up a minority of staff, and nursing assistants (NAs) are the leading workforce in caring for older people [11]. Registered nurses are responsible for developing UI care plans and implementing professional care for residents, such as indwelling urinary catheter care and pelvic floor muscle training. NAs assume responsibility for providing usual care for UI, such as replacing diapers and completing a voiding diary. However, there is a general shortage of NAs in Chinese NHs, and this shortage can negatively impact UI care due to fatigue and heavy workloads [12]. Additionally, NAs often have a low educational level and no professional education regarding UI [13]. According to the knowledge, attitude, and practice (KAP) model, individual practices (behaviours) are determined by their knowledge and attitude [14]. Adult learning theories play an important role in the design of training and education programs, emphasizing the internal motivation of learning and the practicality of knowledge [15]. NAs rarely receive consistent systematic training regarding UI, which makes it challenging to provide high-quality UI care [16].

Studies have found that NAs possess insufficient knowledge about preventing and managing UI and have negative attitudes toward UI care in older people [17, 18]. NAs in Korea who were tested on their UI knowledge had a less than 50% correct response rate, and the mean score in the United Kingdom was 5.5 out of a possible 14 points [17, 19]. The percentage of correct responses of among Taiwanese NAs regarding UI knowledge was only 71%, but they had positive attitudes toward residents with UI [20]. A qualitative study in Australia was conducted to explore the expectations of nursing home staff about what constitutes quality UI care for NH residents [21]. However, there are no published reports about the knowledge, attitudes, and training needs among NAs regarding UI in mainland China.

A mixed-methods design provides a deeper, broader, and more balanced understanding of the results and addresses the limitations of other methods [22]. The aim of the present study was to assess NAs’ current knowledge and attitudes about UI and explore their training needs regarding UI.

## Methods

### Design

**Fig. 1:**
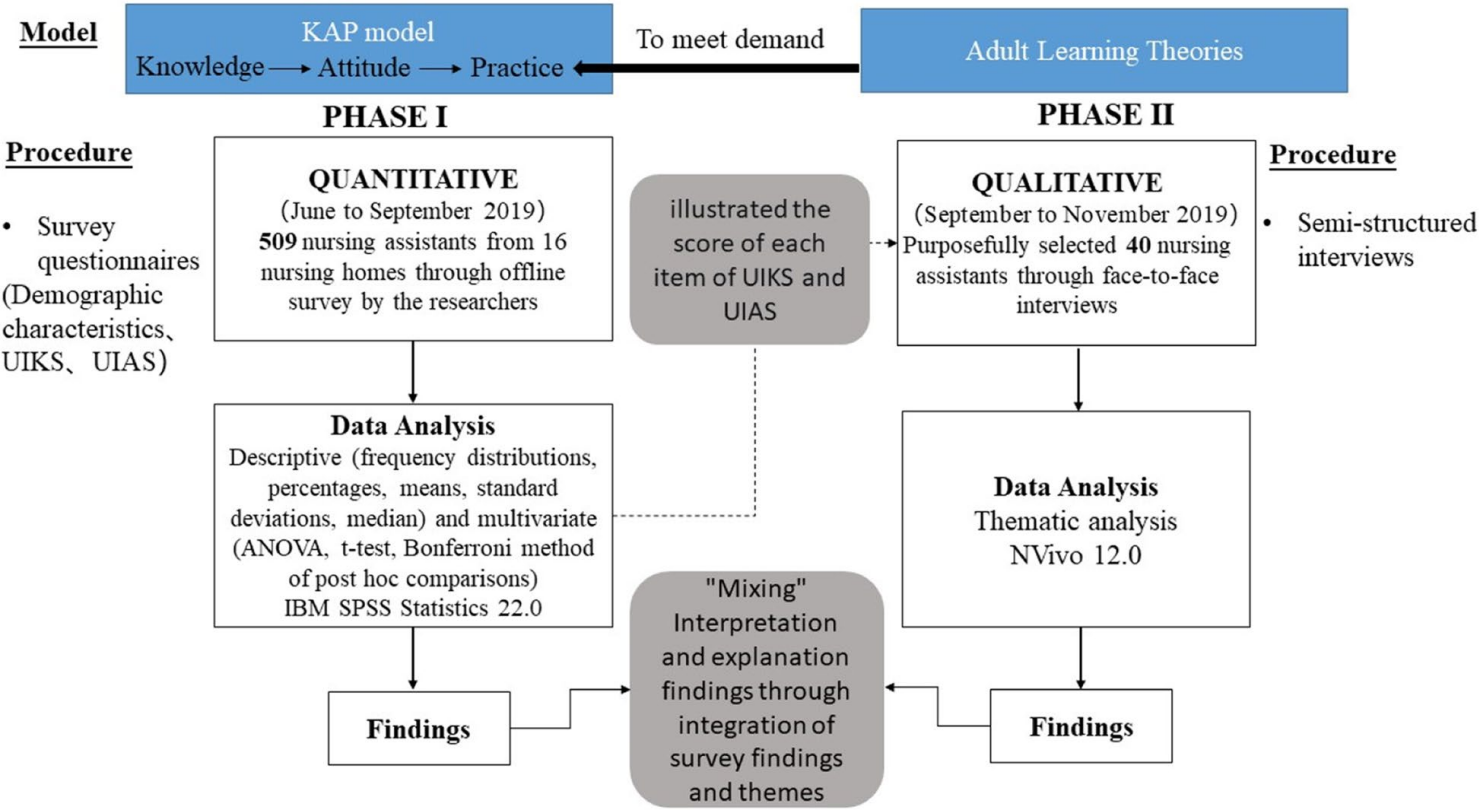
Flowchart of the mixed methods study with an explanatory sequential design UIKS: Urinary Incontinence Knowledge Scale, UIAS: Urinary Incontinence Attitude Scale

In this study, we used a sequential, explanatory, mixed-methods design comprising two phases (Fig. 1) [23]. In Phase I, 509 NAs were randomly selected to complete a survey regarding the knowledge and attitudes of the participants about UI. The results of Phase I provided valuable information to guide semi-structured interviews in Phase II. The researchers integrated qualitative and quantitative data and used qualitative themes to complement and expand the quantitative findings.

**Fig. 2:**
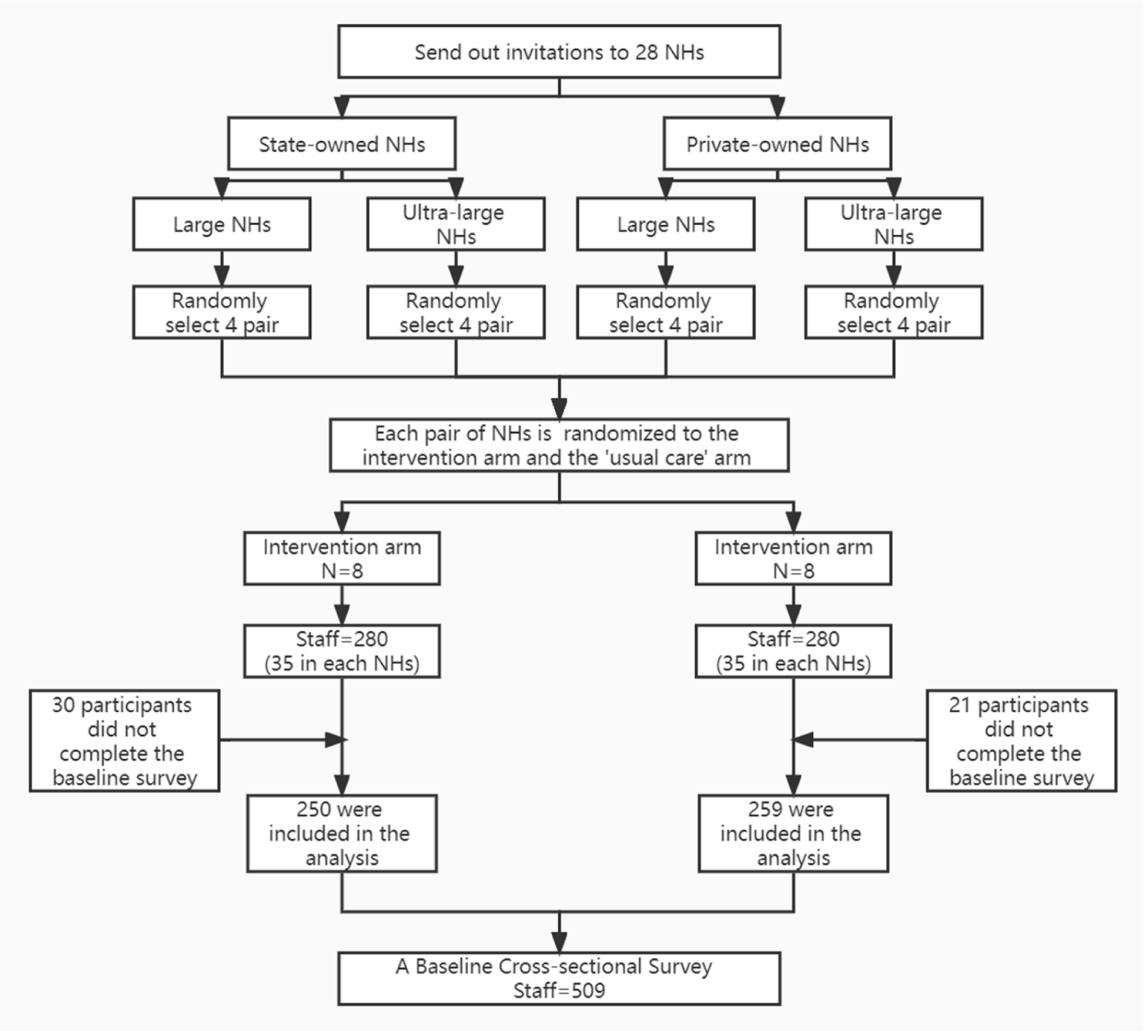
Flowchart of sampling and random assignment. NHs: nursing homes

This study was a part of a cluster randomized controlled trial (RCT) to implement the aged care clinical mentoring model of change in NHs in China. More detailed information about this RCT (e.g., its inclusion and exclusion criteria and sampling method) can be found in our earlier publication [24]. A flowchart of the sampling and random assignment is presented in Fig. 2.

### Participants

NAs were included in this study if they (1) worked in large or very large NHs; (2) had provided direct care for residents with UI for more than 3 months; and (3) volunteered to participate in this study. NAs were excluded from this study if they were also involved in other training and research activities.

### Instruments

The survey questionnaire consisted of demographic characteristics, the Urinary Incontinence Knowledge Scale (UIKS), and the Urinary Incontinence Attitude Scale (UIAS); the UIKS and UIAS were used with permission from the original authors. The demographic information included sex, age, educational level, years of work, experience with UI, the frequency of receiving training on UI, and interest in learning about UI. The UIKS consists of six subscales: (a) risk factors, (b) symptom, (c) impact, (d) prevention, (e) treatment, and (f) management. These subscales included dichotomous items (correct = 1; false or do not know = 0), with scores ranging from 0 to 30 and Cronbach’s alphas ranging from 0.69 to 0.72. The UIAS, developed by Yuan, Williams, and Liu [25], includes 15 items with a 4-point Likert scale used to rank the degree of agreement (strongly disagree, disagree, agree, and strongly agree) with each item. The scores range from 15 to 60, and Cronbach’s alpha ranges from 0.65 to 0.79 [26].

From the perspective of the KAP model and adult learning theories, semi-structured interviews were conducted to explore the beliefs and expectations of NAs regarding UI training. The interview questions were guided by five main topics: knowledge, skills, attitude, resources, and motivation (Table 1).

**Table 1:** Interview questions and corresponding main topics

Main topics	Interview questions
Knowledge	● Can you describe the current state of UI care in the nursing home? ● What do you think NAs need to know about caring for older people with UI?
Skills	● What special skills do you need to improve the quality of care for UI? ● What are the current difficulties in caring for older people with UI, and what can be done to make providing this care easier?
Attitude	● How do you feel about caring for older people with UI? ● What role do you think NAs play in UI care?
Resources	● What resources are currently available to assist you in providing UI care in the nursing home? ● What resources do you need to help you to provide better incontinence care?
Motivation	● Would you be willing to participate in systematic UI education training if given the opportunity? ● Why do you attend UI training? ● What is your motivation for attending UI training?

*UI* urinary incontinence

### Data collection

In Phase I (from June 2019 to September 2019), questionnaires were conducted by three Master of Nursing researchers (LLL, JJJ, and ZYN) to assess NAs’ knowledge and attitudes toward UI. The researchers distributed the questionnaires to NAs at the end of each morning meeting and collected them promptly after completion. This study was conducted in Hunan Province, covering the four prefecture-level cities of Changsha, Xiangtan, Zhuzhou, and Yueyang. A total of 16 NHs were included using cluster random sampling.

In Phase II (from September 2019 to November 2019), focus group interviews were conducted by the researchers (LLL, JJJ, and ZYN). Purposeful sampling was used to identify the participating NHs. According to the inclusion and exclusion criteria and the duty situation on that day, with the help of managers, the researchers selected eight NAs to participate in the interviews. The data reached saturation when interviews were conducted at the fourth nursing home. We conducted interviews at a fifth nursing home to ensure that we had identified all the essential themes. A total of 40 NAs were ultimately included from five NHs. Each participant signed a written informed consent form before the interview. The focus group interviews lasted 40–60 minutes. Before the formal interviews, a pilot study was conducted in one nursing home, and the outline was modified according to the pilot results.

### Statistical analysis

In this study, qualitative and quantitative data were integrated and reported using a narrative approach. In the quantitative survey of Phase I, data were analyzed using IBM SPSS Statistics 22.0 (IBM Corp., Armonk, NY, USA). Descriptive statistics were used to describe the categorical variables and continuous variables (participants’ demographic characteristics). Mean and standard deviation were used to describe UI knowledge and UI attitude scores. Mean comparisons for the continuous variables were performed using independent *t* tests, and tests for categorical variables were performed using chi-square tests. Multiple comparisons were conducted using the Bonferroni method of posthoc comparisons. Statistical significance was set a priori at α = 0.05.

In the qualitative survey of Phase II, interviews were audio-recorded, and the recordings were transcribed for analysis. Thematic analysis was conducted by the researchers (LLL, JJJ, and ZYN) using NVivo 12.0 [27]. During data coding, any disagreements were resolved through discussion until a consensus was reached. The researchers first coded the original data; then the codes were grouped and summarized into themes based on their meaning during repeat processes.

## Results

### Demographics

The participants in the study were mainly female NAs, with 92.1% in Phase I and 95.0% in Phase II. We queried 509 NAs about their knowledge and attitudes toward UI. In total, 16.7% of NAs had a primary school education; 71.7% had a junior high school education; and 11.6% had a high school education. Their demographic information is shown in Table 2. A total of 40 NAs agreed to be interviewed. Their ages ranged from 37 to 65 years, with a length of working time in NHs of 1 to 6 years.

**Table 2:** Demographic information in the quantitative survey (*N* = 509)

Variable	*N*	%
Sex		
Male	40	7.9
Female	469	92.1
Age		
Mean (SD)	50.62 (4.73)	
Range	37–65	
Educational level		
Primary school	85	16.7
Junior high school	365	71.7
High school	59	11.6
Years of work		
< 1	139	27.3
1–2	132	25.9
3–5	102	20.0
> 5	136	26.7
Have you experienced UI yourself?		
Yes	76	14.9
No	433	85.1
The frequency of receiving training on UI before this survey		
Often	90	17.7
Sometimes	220	43.2
Hardly ever	193	37.9
None	6	1.2
Would you like to know more about UI?		
Yes	478	93.9
No	31	6.1

*UI* urinary incontinence, *SD* standard deviation

### Urinary incontinence knowledge

We observed that the overall level of knowledge among NAs about UI care was significantly low. Mean UIKS scores were 14.00 ± 4.18. Among the six dimensions of the UIKS, mean scores for the dimensions of risk factors (1.48 ± 1.04) and treatment (1.33 ± 1.00) were lower than those for the other four dimensions. No NAs achieved a correct response rate of 100%; the highest correct response rate (68.01%) was for the symptom dimension. Only 27.6% of respondents believed that avoiding obesity could reduce the risk of UI, and 69.2% felt that UI was a normal part of aging.

The findings showed a significant difference in knowledge across different years of working experience (F = 52.96, *P* < 0.01; Table 3). NAs with at least 5 years of work experience had better knowledge than NAs with < 1 year (mean difference 5.52, *P* < 0.01), 1–3 years (mean difference 3.25, *P* < 0.01), and 3–5 years (mean difference 2.53, *P* < 0.01) of work experience. In addition, different educational levels were found to be significantly related to knowledge scores (F = 11.061, *P* < 0.01) (Table 4). The UIKS scores of NAs with a high school education were higher than those of NAs with a primary school or junior middle school education.

**Table 3:** Mean UIKS score according to years of work in a nursing home

Mean scores (SD)	Years of work				Maximum obtainable score	F	p	All participants	% Correct answer	Level
	< 1	1-2	3-5	> 5						
Overall score	11.34(4.06)	13.62(3.57)	14.33(3.00)	16.86(3.74)	30	52.957	< 0.001*	14.00(4.18)	46.70	Poor
Risk factors	1.02(0.98)	1.43(0.95)	1.42(0.93)	2.05(1.02)	5	25.419	< 0.001*	1.48(1.04)	29.70	Poor
Symptom	2.61(1.31)	3.45(1.21)	3.59(1.10)	4.00(1.04)	5	33.138	< 0.001*	3.40(1.28)	68.01	Moderate
Impact	2.61(1.16)	2.79(1.25)	2.99(1.14)	3.30(1.25)	5	8.112	< 0.001*	2.92(1.23)	58.39	Poor
Prevalence	1.10(1.34)	1.52(1.49)	1.81(1.30)	2.41(1.46)	5	20.839	< 0.001*	1.70(1.49)	34.07	Poor
Treatment	0.98(1.07)	1.31(0.94)	1.47(0.87)	1.59(1.00)	5	9.700	< 0.001*	1.33(1.00)	26.60	Poor
Management	3.00(0.90)	3.10(0.92)	3.03(0.84)	3.50(0.95)	5	8.763	< 0.001*	3.17(0.93)	63.42	Moderate

*UIKS* Urinary Incontinence Knowledge Scale, *SD* standard deviation

A total score < 60% indicates poor knowledge, 60–80% indicates moderate knowledge, and > 80% indicates good knowledge

**Table 4:** Relationship among Knowledge, Attitudes and Sociodemographic Characteristics

Characteristics	Knowledge			Attitudes		
	Mean (SD)	T/F	p	Mean/SD	T/F	p
Overall score	14.00(4.18)			35.51(3.18)		
Sex						
Male	13.87(4.34)	−0.212	0.832	35.25(2.29)	53.362	0.468
Female	14.02(4.17)			35.53(3.25)		
Educational level						
Primary school	14.74(3.71)	11.061	<0.001*	35.49(3.89)	7.073	0.001*
Junior high school	13.51(4.21)			35.28(2.76)		
High school	16.01(3.90)			36.95(4.05)		
Years of work						
< 1	11.34(4.06)	52.957	<0.001*	35.55(3.06)	2.185	0.089
1-2	13.62(3.57)			35.14(2.74)		
3-5	14.33(3.00)			35.23(3.40)		
> 5	16.86(3.74)			36.05(3.48)		
Have you experienced UI yourself						
Yes	14.63(4.21)	1.406	0.160	35.31(2.67)	−0.590	0.556
No	13.90(4.17)			35.55(3.27)		
The frequency of receiving training on UI before this survey						
Often	14.27(4.13)	1.817	0.143	35.61(3.43)	3.498	0.015*
Sometimes	14.05(4.47)			35.81(3.34)		
Hardly ever	13.73(3.85)			35.04(2.77)		
None	17.50(2.88)			38.16(3.81)		
Would you like to know more about UI?						
Yes	14.24(4.02)	5.048	<0.001*	35.61(3.20)	36.548	0.002*
No	10.42(4.99)			34.00(2.54)		

*p < 0.05

*UI* urinary incontinence

The qualitative findings corroborated the lack of knowledge regarding UI. Many participants stated that the current institutional training focused on daily care, with little training in UI care.

*The content of our daily training courses (issued by the department of civil affairs), mainly includes dietary care, elimination care, and sleep care. Training related to UI only covers general daily procedures of elimination care such as diaper changing and how to help bedridden older people use the bedpan, and there is a lack of specialized training for UI care.* (A2).

### Urinary incontinence attitudes

Most NAs had positive attitudes toward UI (mean score = 35.51, SD = 3.19). Approximately 91.9% (*n* = 468) of NAs thought UI was difficult to talk about because it is an embarrassing problem. Only 16.9% (*n* = 86) of NAs thought UI could be prevented.

There were significant differences in attitudes among NAs with different educational levels (F = 7.073, *P* < 0.01) (Table 4). Different frequencies of training in UI before this investigation were found to be significantly related to attitude scores (F = 3.498, *P* < 0.05) but not to knowledge scores (F = 1.817, *P* = 0.143). In the interviews, many NAs attached great importance to shifting conventional thinking due to a lack of correct perceptions in caring for older people with UI.

*Actually, I think I would be ashamed…[chuckle]…if I had urine leakage. However, as front-line caregivers, we have the most contact with older people, and if we ourselves think urine leakage is normal and shameful, we do not even take it seriously, let alone older people.* (A8).

### Nursing assistants’ learning autonomy regarding urinary incontinence

Among NAs, 93.9% (*n* = 478) indicated that they wanted personalized training in UI care. The intention to learn more about UI was found to be significantly related to knowledge scores (t = 5.048, *P* < 0.001) and attitude scores (t = 36.548, *P* = 0.002). Some participants emphasized the importance of training and identified the factors affecting UI learning, which were described as follows.

1. **Learning motivation**

Participants showed that they were willing to actively participate if they were allowed to learn.

*If I can choose, I would be willing to participate in the UI training. For us, the most frequent UI care operations are replacing the wet pants, diapers, and bedsheets of residents with UI. Our enthusiasm to provide better UI care has been severely weakened...* (A1)

(2) **Low educational background**

Many participants indicated that they generally had a low educational background, which affected their absorption of knowledge.

*…being old, having low education. If you present complex knowledge, it is difficult for us to understand. I hope you simplify the content so that we can understand it. Moreover, the words in the learning manuals were very small for us, so I could not see them clearly.* (A40)

(3) **Time constraints**

Some participants stated that time constraints were an important factor that impeded their learning.

*It would be nice to learn more about UI, but it would hopefully not take up too much of our downtime. As you know, we are swamped. We have to take care of 7–8 older people on our own; the workload is high. If you want to take up our rest time, we will not be happy…*(A7).

### Preferred training styles regarding urinary incontinence

Only 17.7% (*n* = 90) of the NAs frequently received UI training, 43.2% (*n* = 220) sometimes received UI training, 37.9% (*n* = 193) hardly ever received UI training, and 1.2% (*n* = 6) never received UI training. Some participants reported that the previous training was boring and the jargon was too technical for them to understand. The following most preferred training styles were identified.

1. **Face-to-face guidance from a mentor**

Some NAs preferred face-to-face guidance from a mentor to improve their professional skills.

*I like face-to-face guidance because I can quickly understand what I did wrong, which I think is more valuable than what you tell me in front of the computer. In this way, I can apply what they teach me directly to clinical work.* (A20)

(2) **Training combining theory with practice**

Some participants advocated the combination of theory and practice, believing that it could better translate knowledge into practice.

*If you only teach us theoretical knowledge, we will not understand easily. The method of theory and practice are combined, making dull content easier to understand. In addition, I think more clinical practice skills could be taught, because they are more practical than theoretical knowledge.* (A25)

(3) **Online video training**

Many participants stated that they enjoyed learning with online videos, which they perceived as a more convenient option.

*We can download the videos onto our mobile phones. In our free time, we can watch videos repeatedly. In addition, the content cannot be remembered over the long term, only in short-term memory. However, if we learn with video, we can watch it again and again at any time, and we will be more influenced.* (A33).

## Discussion

Our study aimed to explore NAs’ knowledge, attitudes, and training needs regarding UI through a mixed-methods approach [28]. The results showed that Chinese NAs have low UI knowledge but positive attitudes toward UI care. These results are congruent with findings from Norway, Turkey, and Taiwan [20, 29, 30]. We also found that many NAs would like more training in UI, but only a few NAs were regularly offered training. Mathis (2014) noted that evidence-based education and training programs can improve NAs’ knowledge about UI and change their attitudes toward UI [31]. However, little attention has been directed toward UI training for NAs among NHs in China.

The results of our study showed that the knowledge level among NAs in NHs in China is low. Less than one-third of NAs believed that avoiding obesity could reduce the risk of UI, and nearly 70% thought that UI is a normal part of aging. Notably, mastery of UI-related knowledge and correct care concepts are the basis for providing effective UI care. It has been suggested that correct guidance and encouragement from health professionals are beneficial in encouraging older people to access timely treatment [32]. Another important finding is that NAs with longer work experience and higher educational backgrounds had higher UIKS scores. This result was in accordance with a previous study indicating similar correlations between educational background and knowledge among NAs [33]. These findings highlight the importance of prioritizing the establishment of senior and highly educated NAs as supervisors to guide less experienced NAs.

The most obvious finding is that despite their poor knowledge scores, NAs had generally positive attitudes toward UI care. A previous study revealed that fewer registered nurses had positive attitudes than NAs, although the level of UI knowledge among registered nurses was superior to that among NAs [20]. Importantly, UI care is not only the responsibility of only NAs or only registered nurses. Appropriate team collaboration between registered nurses and NAs in daily practice is necessary [20]. Similar to another study [34], we found no statistically significant difference in UI attitude scores by sex. NAs with a high school education and those with more UI training experience had more positive attitudes than those with primary school education and those who had less training experience. These results are inconsistent with the findings of another study showing that educational background did not influence nurses’ attitudes toward UI [35]. This may also be related to the higher educational level of registered nurses, in general.

The results of the present study showed that more than 90% of NAs were eager to receive training in UI. Learning autonomy about UI care was found to be significantly associated with UI knowledge and attitudes. Motivation is the psychological tendency or internal drive that stimulates and maintains and directs a person’s action toward a certain goal [36]. Based on the Capability, Opportunity, and Motivation Behaviour model (COM-B model), capabilities (knowledge and attitude) can affect intrinsic motivation, and motivation plays an important role in behaviour change [37]. However, our results showed that NAs considered low educational background and time constraints to be barriers to successful training. A previous study demonstrated that implementing person-centered care training in long-term care settings also involves time constraints [38]. Therefore, convenient teaching materials and personalized learning modules relevant to clinical practice are vital to boosting enthusiasm for participation [39].

In this study, NAs had three preferred training styles: face-to-face guidance from a mentor, training combining theory with practice, and online video training. UI training programs in NHs in the United States include 4-hour online training sessions. The project staff provides offline guidance and feedback to caregivers at a specific time [31]. The appointment of clinical mentors as a means of QI has been widely reported and is recognized as a successful model by geriatric care agencies around the world [40, 41]. Facility managers should select appropriate registered nurses as mentors to train NAs, and a senior NA can be selected as the site leader to supervise junior NAs [42]. A previous study indicated that even after educational intervention, NAs did not change their concepts regarding UI and aging, which may indicate that training was not sufficiently practical or in-depth [43]. Accordingly, theoretical knowledge should be combined with practical skills. Typical learning via case sharing and operation manuals with graphics and text are necessary, and hands-on learning techniques are vital to long-term memory retention [44].

As a crucial health problem, UI is a valid indicator of care quality in NHs [45]. Effective UI care could result in significant health benefits for NH residents with UI. To improve the care quality of UI in NHs, developing a reasonable and effective UI training program is crucial for first-line NAs.

## Limitations

This study has several limitations. First, our sample was selected from 16 NHs in Hunan Province; therefore, it is not representative of the entire population of NAs in Chinese NHs, which may limit the generalizability of our results. Second, when selecting interviewees with the help of managers, managers are likely to give preference to NAs who are more active in their daily work. In addition, it must be acknowledged that qualitative data analysis can be influenced by the researchers’ perspectives. However, we performed strict quality control in the process of analyzing the data in the qualitative study phase. Last, we did not obtain additional qualitative information from the perspective of multiple stakeholders. In the future, researchers should consider this issue to obtain richer descriptions.

## Conclusions

The present study’s findings clearly indicated that Chinese NAs in NHs generally had positive attitudes toward UI, despite their limited knowledge of UI. NAs had strong learning autonomy, but low educational backgrounds and time constraints were barriers to learning enthusiasm. In light of this, diversified training styles should be adopted according to the actual situation of NHs. It is essential for facility managers to develop training and learning mechanisms for UI in daily work. Further research to construct a targeted UI training program is important to raise awareness of effective incontinence management and to improve the quality of life among NH residents with UI.

## Data Availability

The datasets used or analszed during the current study are available from the corresponding author upon reasonable request.

## Abbreviations

UI: Urinary incontinence
QI: Quality improvement
NHs: Nursing homes
NAs: Nursing assistants
KAP model: Knowledge, Attitude and Practice model
COM-B model: Capability-Opportunity-Motivation-Behaviours model
RCT: Randomized Controlled Trial
UIKS: Urinary Incontinence Knowledge Scale
UIAS: Urinary Incontinence Attitude Scale

## References

[1] Milsom I, Gyhagen M (2019). The prevalence of urinary incontinence. Climacteric.

[2] Tai H, Liu S, Wang H, Tan H (2021). Determinants of urinary incontinence and subtypes among the elderly in nursing homes. Front Public Health.

[3] Kwong PW, Cumming RG, Chan L, Seibel MJ, Naganathan V, Creasey H, Le Couteur D, Waite LM, Sambrook PN, Handelsman D (2010). Urinary incontinence and quality of life among older community-dwelling Australian men: the CHAMP study. Age Ageing.

[4] Chang OH, Hacker MR, Rosenblatt PL, Neo D, Von Bargen E, Berrahou I, Le A, Lefevre R, Hota LS (2019). Comparing postoperative voiding dysfunction after mid-urethral sling using either a Babcock or Kelly clamp tensioning technique. Int Urogynecol J.

[5] Vethanayagam N, Orrell A, Dahlberg L, McKee KJ, Orme S, Parker SG, Gilhooly M (2017). Understanding help-seeking in older people with urinary incontinence: an interview study. Health Soc Care Community.

[6] Xu D, Liu N, Qu H, Chen L, Wang K (2016). Relationships among symptom severity, coping styles, and quality of life in community-dwelling women with urinary incontinence: a multiple mediator model. Qual Life Res.

[7] Cacciari LP, Kouakou CR, Poder TG, Vale L, Morin M, Mayrand MH, Tousignant M, Dumoulin C (2022). Group-based pelvic floor muscle training is a more cost-effective approach to treat urinary incontinence in older women: economic analysis of a randomised trial. J Physiother.

[8] Voegeli D, Hillery S (2021). Prevention and management of moisture-associated skin damage. Br J Nurs.

[9] Huang P, Luo K, Wang C, Guo D, Wang S, Jiang Y, Huang W, Zhang W, Ding M, Wang J (2021). Urinary incontinence is associated with increased all-cause mortality in older nursing home residents: a Meta-analysis. J Nurs Scholarsh.

[10] Kampstra NA, Zipfel N, van der Nat PB, Westert GP, van der Wees PJ, Groenewoud AS (2018). Health outcomes measurement and organizational readiness support quality improvement: a systematic review. BMC Health Serv Res.

[11] Lindh Falk A, Hult H, Hammar M, Hopwood N, Abrandt Dahlgren M (2018). Nursing assistants matters-an ethnographic study of knowledge sharing in interprofessional practice. Nurs Inq.

[12] Parra-Anguita L, García-Fernández FP, Del-Pino-Casado R, Pancorbo-Hidalgo PL (2019). Knowledge about the Care of People with Alzheimer's disease of the nursing staff of nursing homes in Spain. Int J Environ Res Public Health.

[13] Liu M, Lam B, Fong P, Yuan HB. Nursing shortage: the facts and strategies in Macao society. Online J Issues Nurs. 2012;18(1). 10.3912/OJIN.Vol18No01PPT02. 10.3912/OJIN.Vol18No01PPT02 23452194

[14] Alzghoul BI, Abdullah NA (2015). Pain management practices by nurses: an application of the knowledge, attitude and practices (KAP) model. Glob J Health Sci.

[15] Mukhalalati BA, Taylor A (2019). Adult learning theories in context: a quick guide for healthcare professional educators. J Med Educ Curric Dev.

[16] Palmer MH (2008). Urinary incontinence quality improvement in nursing homes: where have we been? Where are we going?. Urol Nurs.

[17] Park S, De Gagne JC, So A, Palmer MH (2015). Knowledge, attitudes, beliefs, and practices in registered nurses and care aids about urinary incontinence in Korean nursing homes: a cross-sectional survey. J Wound Ostomy Continence Nurs.

[18] Ostaszkiewicz J, Tomlinson E, Hunter K (2020). The effects of education about urinary incontinence on Nurses' and nursing Assistants' knowledge, attitudes, continence care practices, and patient outcomes: a systematic review. J Wound Ostomy Continence Nurs.

[19] Sackley CM, Rodriguez NA, van den Berg M, Badger F, Wright C, Besemer J, van Reeuwijk KT, van Wely L (2008). A phase II exploratory cluster randomized controlled trial of a group mobility training and staff education intervention to promote urinary continence in UK care homes. Clin Rehabil.

[20] Lin SY, Wang RH, Lin CC, Chiang HY (2012). Competence to provide urinary incontinence care in Taiwan's nursing homes: perceptions of nurses and nurse assistants. J Wound Ostomy Continence Nurs.

[21] Ostaszkiewicz J, Tomlinson E, Hutchinson AM (2018). "dignity": a central construct in nursing home staff understandings of quality continence care. J Clin Nurs.

[22] Simonovich S (2017). The value of developing a mixed-methods program of research. Nurs Sci Q.

[23] Schoonenboom J, Johnson RB (2017). How to construct a mixed methods research design. Kolner Z Soz Sozpsychol.

[24] Feng H, Li H, Xiao LD, Ullah S, Mao P, Yang Y, Hu H, Zhao Y (2018). Aged care clinical mentoring model of change in nursing homes in China: study protocol for a cluster randomized controlled trial. BMC Health Serv Res.

[25] Yuan HB, Williams BA (2010). Knowledge of urinary incontinence among Chinese community nurses and community-dwelling older people. Health Soc Care Community.

[26] Yuan HB, Williams BA, Liu M (2011). Attitudes toward urinary incontinence among community nurses and community-dwelling older people. J Wound Ostomy Continence Nurs.

[27] Braun V, Clarke V (2014). What can "thematic analysis" offer health and wellbeing researchers?. Int J Qual Stud Health Well-being.

[28] Younas A, Pedersen M, Tayaben JL (2019). Review of mixed-methods research in nursing. Nurs Res.

[29] Vinsnes AG, Harkless GE, Haltbakk J, Bohm J, Hunskaar S (2001). Healthcare personnel's attitudes towards patients with urinary incontinence. J Clin Nurs.

[30] Yenisehir S, Karakaya IC, Karakaya MG (2019). Knowledge and practice of nursing home caregivers about urinary incontinence. Eur Geriatr Med.

[31] Mathis S, Ehlman K, Dugger BR, Harrawood A, Kraft CM (2013). Bladder buzz: the effect of a 6-week evidence-based staff education program on knowledge and attitudes regarding urinary incontinence in a nursing home. J Contin Educ Nurs.

[32] Yan F, Xiao LD, Zhou K, Li Z, Tang S (2022). Perceptions and help-seeking behaviours among community-dwelling older people with urinary incontinence: a systematic integrative review. J Adv Nurs.

[33] Saxer S, de Bie RA, Dassen T, RJG H (2009). Knowledge, beliefs, attitudes, and self-reported practice concerning urinary incontinence in nursing home care. J Wound Ostomy Continence Nurs.

[34] Cheng WL-S, Kam MK, Liong YY, Tang TC, Tse EHL, Tse HK, Tsao WH, Cheung KC (2022). Factors influencing nursing Students' knowledge of and attitudes toward urinary incontinence. J Wound Ostomy Continence Nurs.

[35] McCann M, Kelly AM, Eustace-Cook J, Howlin C, Daly L (2022). Community nurses' attitudes, knowledge and educational needs in relation to urinary continence, continence assessment and management: a systematic review. J Clin Nurs.

[36] Ryan RM, Deci EL (2000). Intrinsic and extrinsic motivations: classic definitions and new directions. Contemp Educ Psychol.

[37] Michie S, van Stralen MM, West R (2011). The behaviour change wheel: a new method for characterising and designing behaviour change interventions. Implement Sci.

[38] Viau-Guay A, Bellemare M, Feillou I, Trudel L, Desrosiers J, Robitaille MJ (2013). Person-centered care training in long-term care settings: usefulness and Facility of Transfer into practice. Can J Aging.

[39] Terzoni S, Montanari E, Mora C, Destrebecq A (2011). Urinary incontinence in adults: nurses' beliefs, education and role in continence promotion. A narrative review. Arch Ital Urol Androl.

[40] Moyle W, Venturato L, Cooke M, Hughes J, van Wyk S, Marshall J (2013). Promoting value in dementia care: staff, resident and family experience of the capabilities model of dementia care. Aging Ment Health.

[41] Joo JH, Hwang S, Abu H, Gallo JJ (2016). An innovative model of depression care delivery: peer mentors in collaboration with a mental health professional to relieve depression in older adults. Am J Geriatr Psychiatry.

[42] Davila H, Abrahamson K, Mueller C, Inui TS, Black AG, Arling G (2016). Nursing assistant perceptions of their role in quality improvement processes in nursing homes. J Nurs Care Qual.

[43] Hunter KF, Dahlke S (2021). Nurse and health care aide knowledge of urinary continence promotion and management in hospitalized older people. J Wound Ostomy Continence Nurs.

[44] Holm MR, Burkhartzmeyer HL, Fort R, Rinville R, Sannon H (2020). The long-term impact of providing nursing education in a low-income country. Public Health Nurs.

[45] Burke RE, Werner RM (2019). Quality measurement and nursing homes: measuring what matters. BMJ Qual Saf.

