# Correction to: Nursing assistants’ knowledge, attitudes and training needs regarding urinary incontinence in nursing homes: a mixed-methods study

**DOI:** 10.1186/s12877-023-04108-5

**Published:** 2023-07-27

**Authors:** Lulu Liao, Hui Feng, Jingjing Jiao, Yinan Zhao, Hongting Ning

**Affiliations:** 1 School of Nursing, Tongji Medical College, Huazhong University of Science and Technology, Wuhan, China; 2 Xiangya School of Nursing, Central South University, Changsha, China; 3 Xiangya-Oceanwide Health Management Research Institute, Central South University, Changsha, China; 4 National Clinical Research Center for Geriatric Disorders, Xiangya Hospital, Changsha, China

**Correction to: BMC Geriatrics (2023) 23:39**

10.1186/s12877-023-03762-z

After publication of this article [1], the authors reported that the first affiliation of the first author should be removed, leaving the second one.

The original article [1] has been corrected.
